# Types and Frequency of Infusion Pump Alarms and Infusion-Interruption to Infusion-Recovery Times for Critical Short Half-Life Infusions: Retrospective Data Analysis

**DOI:** 10.2196/14123

**Published:** 2019-08-12

**Authors:** James Waterson, Arkadiusz Bedner

**Affiliations:** 1 Medication Management Solutions Becton Dickinson Limited Eysins Switzerland

**Keywords:** medical device, infusion pump, alarm fatigue, critical infusions, alarm management, event log, infusion continuity, critical care, critical short half-life infusions, patient safety

## Abstract

**Background:**

Alarm fatigue commonly leads to a reduced response to alarms. Appropriate and timely response to intravenous pump alarms is crucial to infusion continuity. The difficulty of filtering out critical short half-life infusion alarms from nonurgent alarms is a key challenge for risk management for clinicians. Critical care areas provide ample opportunities for intravenous medication error with the frequent administration of high-alert, critical short half-life infusions that require rigorous maintenance for continuity of delivery. Most serious medication errors in critical care occur during the execution of treatment, with performance-level failures outweighing rule-based or knowledge-based mistakes.

**Objective:**

One objective of this study was to establish baseline data for the types and frequency of alarms that critical care clinicians are exposed to from a variety of infusion devices, including both large volume pumps and syringe drivers. Another objective was to identify the volume of these alarms that specifically relate to critical short half-life infusions and to evaluate user response times to alarms from infusion devices delivering these particular infusions.

**Methods:**

The event logs of 1183 infusion pumps used in critical care environments and in general care areas within the European region were mined for a range of alarm states. The study then focused on a selection of infusion alarms from devices delivering critical short half-life infusions that would warrant rapid attention from clinicians in order to avoid potentially harmful prolonged infusion interruption. The reaction time of clinicians to infusion-interruption states and alarms for the selected critical short half-life infusions was then calculated.

**Results:**

Initial analysis showed a mean average of 4.50 alarms per infusion in the general critical care pump population as opposed to the *whole hospital* rate of 1.39. In the pediatric intensive care unit (PICU) group, the alarms per infusion value was significantly above the mean average for all critical care areas, with 8.61 alarms per infusion. Infusion-interruption of critical short half-life infusions was found to be a significant problem in all areas of the general critical care pump population, with a significant number of downstream (ie, vein and access) occlusion events noted. While the mean and median response times to critical short half-life infusion interruptions were generally within the half-lives of the selected medications, there was a high prevalence of outliers in terms of reaction times for all the critical short half-life infusions studied.

**Conclusions:**

This study gives an indication of what might be expected in critical care environments in terms of the volume of general infusion alarms and critical short half-life infusion alarms, as well as for clinician reaction times to critical short half-life infusion-interruption events. This study also identifies potentially problematic areas of the hospital for alarm fatigue and for particular issues of infusion and infusion-line management. Application of the proposed protocols can help create benchmarks for pump alarm management and clinician reaction times. These protocols can be applied to studies on the impact of alarm fatigue and for the evaluation of protocols, infusion-monitoring strategies, and infusion pump-based medication safety software aimed at reducing alarm fatigue and ensuring the maintenance of critical short half-life infusions. Given the frequency of infusion alarms seen in this study, the risk of alarm fatigue due to the white noise of pump alarms present in critical care, to which clinicians are constantly exposed, is very high. Furthermore, the added difficulties of maintaining critical short half-life infusions, and other infusions in specialist areas, are made clear by the high ratio of *downstream occlusion* to *infusion starts* in the neonatal intensive care unit (NICU). The ability to quantitatively track the volume of alarms and clinician reaction times contributes to a greater understanding of the issues of alarm fatigue in intensive care units. This can be applied to clinical audit, can allow for targeted training to reduce nuisance alarms, and can aid in planning for improvement in the key area of maintenance of steady-state plasma levels of critical short half-life infusions. One clear conclusion is that the medication administration *rights* should be extended to include *right maintenance* and ensured delivery continuity of critical short half-life infusions.

## Introduction

### Background

Alarm fatigue is the presence of “frequent false alarms, leading to a reduced response to alarms” [1] and is caused by both false and nonactionable alerts. It can cause sleep disturbances, impaired healing, and intensive care unit (ICU) delirium for patients and contributes to staff burnout [2].

Infusion pumps are only one of numerous medical devices that are present at the bedside of critically ill adults, children, and neonates. Like other devices, there is a strong probability that their alarms may be ignored or at least *downgraded* in terms of the requirement for an immediate response by staff who are exposed to an excessive number of alarms, many of which are false, clinically insignificant, and essentially nonactionable [3]. Indeed, one study found that only 15% of cardiovascular alarms in an ICU setting were clinically relevant [4].

While alarm fatigue as a subject does embrace the alarms from all bedside medical devices, it is pertinent to focus on infusion pumps, as critical care patients commonly receive multiple infusions of sedatives, muscle relaxants, parental nutrition, fluid maintenance, antibiotics, and antivirals, as well as critical short half-life infusions. This means that the problem of *white noise* carries extra complications for these devices. Pump alarms first need to be separated out from other device alarms such as those from ventilators, monitors, and even low-grade technical alarms from beds or warming blankets. Alarms also need to be quickly rated for *immediate response required* as in the case of critical short half-life medications, where any infusion interruption can have potentially catastrophic hemodynamic consequences for the patient. These critical alarms requiring immediate response are only some of many other *suitable nonimmediate response required* alarms experienced by the clinician. For example, an alert for *near end of infusion* (NEOI) or *end of infusion* (EOI) requires an immediate response if the medication is a critical short half-life infusion but takes a lower priority if the alert is for a medication such as ganciclovir or amoxycillin completing a dose.

The issue at hand is that such filtering is extremely difficult with multiple infusions at the bedside and the problem is compounded when nurses are caring for more than one patient. This is made more complex by the common need to balance acuity in the nursing staff’s respective workload; giving each nurse an equal acuity load may require geographical convenience to be sacrificed. The increasingly common use of isolation rooms in critical care areas for infection control and protection of the patient also makes direct visualization of the patient and pumps increasingly difficult. Even beyond critical care, infusion therapy is one of the most widely practiced therapies in any health care organization, with alarms in the general hospital pump population accounting for 5% of lost infusion time per year [5].

A number of previous studies have focused on *time and motion* outcomes, device management, and equipment maintenance processes to reduce the number of alarms from pumps [5]. General reaction times have been recorded in one study where mean resolution times for 83% of alarms were 1 minute or less, but outliers of some 3% of alarms were not dealt with for more than 4 minutes [6].

Those studies are certainly of value and this study continues this work with a large-scale examination of alarm types. Added to this is the metric of reaction time for critical short half-life infusions and ready-made code for interrogation of the event logs of specific pumps.

### Objectives

The overall objective of this study was to establish baseline data for volume of alarms and to note differences across the various environments that fall under critical care and within the data available to us from the following hospital units: pediatric intensive care unit (PICU), neonatal intensive care unit (NICU), general (adult) intensive care unit (GICU), and coronary (adult) intensive care unit (CICU). Each of these areas faces different challenges in terms of the complexity of infusion therapy, intravenous access devices used, patient characteristics, and staffing patterns.

In terms of critical short half-life infusion management and patient safety, the key measurements undertaken in the study included metrics to assist in an assessment of the ability of clinicians to differentiate between infusion-interruption alarms for these medications and other nonurgent alarms and to then act quickly to resolve infusion interruptions. The study’s objectives fell into two high-level processes:

1. Identifying the overall volume of pump alarms experienced versus the number of these alarms that apply to critical short half-life infusions is of value for the assessment of the background of *white noise*, against which clinicians are operating on a daily basis in the ICU environment.
2. Identifying clinician reaction time to critical short half-life infusion alarms and time to resolution of the issue.

The above information and the creation of protocols for interrogating pump event logs to gain this data was felt to be of value going forward for studies into strategies that could reduce spurious alarms, make prioritization of alarms easier, and improve reaction times to critical short half-life infusion interruption. In this respect, the value of measuring the total volume of alarms experienced by clinicians in critical care is a key parameter, as reducing this number overall would be expected to directly impact on the second measured parameter: reaction time to critical short half-life infusion interruption.

## Methods

### Study Design

This retrospective study was conducted on the anonymized event logs of 1183 infusion pumps—566 CareFusion, Becton, Dickinson and Company (BD) Alaris Plus and Guardrails Plus large volume pumps (LVPs) and 617 syringe pumps (SPs)—used in critical care environments within the European region over a period of 6482 days, between January 1, 2000, and September 29, 2017.

Pumps were of various ages and carried a range of dose error reduction software (DERS) solutions; in all cases, the pump’s event log was bundled with near-miss medication error reporting data (ie, BD Alaris continuous quality improvement [CQI] software).

DERS software is generally comprised of two parts: (1) a drug library that contains the names of medications to be given by infusion, with minimum and maximum doses for each medication, and (2) a dataset that contains the drug library and has default configurations for the pump, including alarm settings such as maximum occlusion pressure, air-in-line threshold, and alarm volume. The features of the DERS used by the study pumps are given below.

The event logs were mined via SQL Server Management Studio v17.9.1 (Microsoft) for a range of alarm states. The fact that DERS was being used extensively by the end users for continuous infusions was vital to the study, as critical short half-life infusions, such as adrenaline/epinephrine, noradrenaline/norepinephrine, dobutamine, dopamine, and glycerol-trinitrate/nitroglycerine (GTN/NTG), could be clearly identified. In the study group, the DERS software was activated for infusions in 67.39% of cases. This percentage of infusions started from within the library can be expected to increase significantly in the near future with the ability to push datasets via wired and wireless systems to all pumps across a facility. This will allow for more complete infusion data, not just from ICUs but also from dispersed locations, including clinics and off-campus sites [7]. This will give more complete data on infusion types, generally, and on critical short half-life infusions and event logs, in particular.

The DERS in use on the study pumps allowed for the creation of distinct profiles; this involved *free-text* entry and it was required to group similar entities to create four distinct types of critical care units for the purposes of comparison and contrast: (1) PICU, (2) CICU, (3) GICU, and (4) NICU. As examples of *similar entities*, intensive care nurseries (ICNs) and special care baby units (SCBUs) were bundled into NICU, and CICUs were bundled with coronary care units (CCUs).

For the purposes of the study, the following critical short half-life infusions were selected: (1) adrenaline/epinephrine, (2) noradrenaline/norepinephrine, (3) dobutamine, and (4) dopamine. These critical short half-life infusions were selected due to their importance and extensive use in all areas of critical care, their potential for patient harm if any interruption to infusion is extended, and the fact that their plasma half-lives have been identified. Free-text entry from drug names, as well as generic terminology, and the differing languages present in the European region also required study grouping of the critical short half-life infusions into generic medication names (see Table 1). GTN/NTG was initially included in the study group, but once data interrogation began it was found that, while it had fairly extensive use in CICUs, its use did not extend across all critical care areas to a sufficient degree. It was therefore excluded from the study.

**Table 1:** Critical short half-life medications with plasma half-lives and sequelae to extended infusion interruption.

Critical short half-life infusion	Plasma half-life in seconds	Possible impact of infusion interruption	Possible impact of postocclusion bolus
Adrenaline/epinephrine	180	Hemodynamic instability and severe hypotension	Hemodynamic instability and severe hypertension Coronary artery contraction
Dobutamine	120	Hemodynamic instability and hypotension Cardiac shock	Hemodynamic instability and hypertension
Dopamine	60-120	Hemodynamic instability and hypotension	Hemodynamic instability and severe hypertension
Noradrenaline/norepinephrine	120-180	Hemodynamic instability and severe hypotension	Hemodynamic instability and severe hypertension Coronary artery contraction

### Infusion Device Types Included in the Study

The LVPs included in the study were CareFusion, BD Alaris Plus and Guardrails Plus general purpose and variable pressure pumps. The SPs included in the study were CareFusion, BD Alaris Plus and Guardrails Plus critical care and general hospital pumps. All of these pumps carry a common DERS, which applies *soft* and *hard* limits to all infusions delivered using the drug library. Soft Limits give the clinician a warning whenever a programmed administration rate or total dose is above or below an accepted dose range. This limit can be overridden by the clinician, thus allowing for clinical judgement to be employed, but only after an advisory message has been given by the DERS and acknowledged by the clinician. Hard limits apply to maximum doses above which the drug would be toxic or where patient harm is likely. Hard limits require the clinician to reprogram a safe dose or rate. All soft and hard limit *breaches* are recorded by the pump. These *flagged-up,* near-miss events are recorded in a CQI reporter log, which can be downloaded directly from the pump or sent directly to the BD Alaris Communication Engine server for connected pumps. The pump’s event log is bundled with this CQI data.

The critical care and general hospital SPs include features designed to maintain infusion continuity and to promote steady plasma levels of infused medications. Both have *Fast Start* [8], which guarantees uptake of mechanical slack (ie, 95% take-up has been achieved by the best-performing pumps) [8], and a highly accurate volumetric purge function [9]. Both also carry a *Back Off* feature, which briefly draws back on the plunger if the occlusion alarm is activated, thereby reducing the size of any postocclusion bolus.

The critical care SP and variable pressure LVP also have in-line pressure monitoring, which is capable of detecting 1 mmHg increments of change in downstream pressure and is monitored via a diaphragm incorporated into the infusion line. This level of accuracy is important to this study as a sensitive, accurate, and rapid time to alarm, since any downstream occlusion is to be expected when these pumps are used for critical short half-life infusion delivery.

The international and independent medical device evaluator KLAS [10] has rated currently available commercial syringe drivers that have a particular focus on factors that reduce the risk of extravasation injury and harm to patients, and that help maintain infusion delivery continuity. These factors were as follows:

1. Shortening the time to alarm for occlusions.
2. Accurately monitoring in-line pressure as a proactive antiextravasation measure.
3. Reducing delays of vasoactive medications at infusion start-up.
4. Decreasing inadvertent bolus following occlusion release.

The above technology would be expected to significantly improve steady-state infusion recovery times for critical short half-life infusions from any interruption to flow, where:

Total infusion recovery time = Time to alarm + Reaction time + Resolution (1)

This is the case, as time to alarm would be shortened by the presence of in-line pressure monitoring, and the resolution phase would be shortened by the *Back Off* and *Fast Start* features.

Taken from the above, the following is the case:

Total noninfusion time = Time to alarm + Reaction time (2)

**Figure 1:**
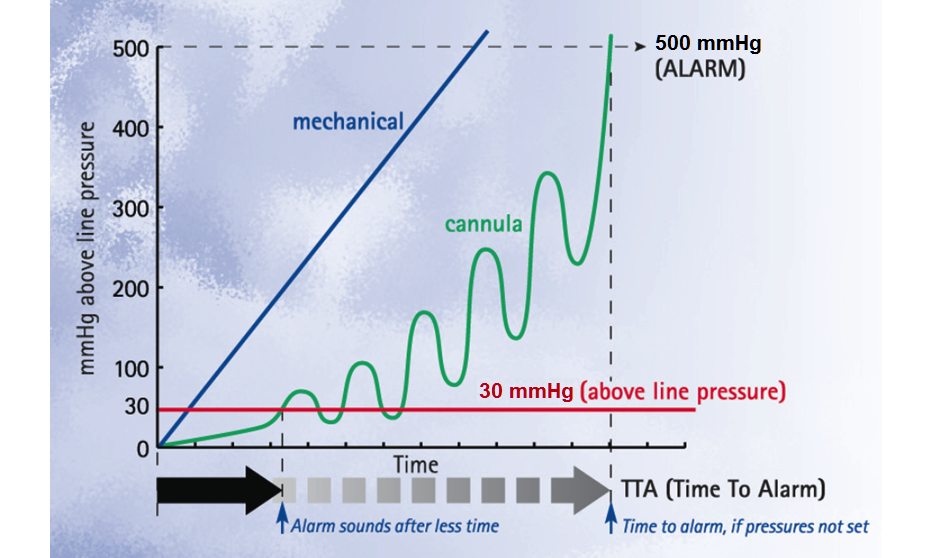
Time to alarm for dynamic alarm pressure setting and a fixed alarm setting for cannula-related and mechanical downstream occlusions.

This of course is also dependent on accurate and appropriate alarm setting [11]. The critical care SP and variable pressure LVP studied here also feature *Auto-offset*, which automatically sets the alarm pressure to a preset level above recorded line pressure and with *Auto-set* pressure, which will automatically set this limit at a predetermined time after the infusion begins. Both of these features are useful in detecting early changes related to vein infiltration and occlusion, and it is generally suggested that the *Auto-offset* be set at 30 mmHg above line pressure [11]. The reason for this is that high-set alarm pressures will delay the alarm even though the patient’s vein is already starting to be in *distress* or there is a hard occlusion, such as a clamp being inadvertently left closed (see Figure 1).

Reaction time is dependent on the clinician being alerted, prioritizing the alarm, responding, and resolving the issue. In many ways, this is the weak link in the chain for critical short half-life infusion maintenance, as even with pumps that carry technology capable of generating an appropriate time to alarm and with appropriately set alarms, resolution of the issue and re-establishment of the infusion is still dependent on the clinician responding to the alarm. Hence our desire to study reaction time in depth and to isolate it as a distinct event and issue for which solutions can be offered for extended and clinically unacceptable reaction times.

The pumps in the study group carry the same pattern of alarm priority across both LVPs and SPs. These generate both audible and visual alarms; therefore, proximity to the alarm is a key factor in the initial recognition of the alarm state. The process of prioritization is assisted by a high-medium-low-normal scheme (see Table 2).

Note that at this *bedside* level, there is no alarm differentiation between drug types (ie, a low-acuity medication alarm for *occlusion* would audibly and visually go off in the same way as a critical short half-life infusion would with the same issue). This is one aspect of the issue of alarm fatigue or “crying wolf” [12].

**Table 2:** Alarm notifications by priority.

Status	Pump beacon color	Alarm tone
High priority	Red	Rapid high pitch
Medium priority	Amber	Rapid low pitch
Low priority	Amber	Slow low pitch
Condition normal	Green	Nil

### Database, Infusion Data Type, and Interface

The drug libraries of the study pumps have the capacity to hold 30 care areas or profiles and have the capacity for 3000 customizable drug types to be created in their respective libraries. As discussed above, the DERS has hard and soft limits for drug dosing and for patient weight, drug concentration, and drug boluses, as well as the ability to set specific occlusion alarm limits for each drug with the critical care and variable pressure pumps.

Any breach of these hard or soft limits is recorded, as well as total drug use across all pumps and compliance with drug library use in the CQI database. Data may be either manually loaded into these databases by a serial connection of pumps to client PCs or aggregated via a real-time infusion data collection service (ie, BD Alaris Communication Engine server software).

### Study Procedure

The data were already patient anonymized, as no personal data such as hospital number, gender, name, data of birth, or other identifiable data were recorded or held on the pumps’ CQI logs or event logs. A further process of anonymization took place with no queries made for institution or facility. Only generic names of profiles or care areas remained.

### Inclusion Criteria

Profiles or care units needed to be grouped into one of the following groups, in order to be included in the study: CICU, NICU, PICU, or GICU. For the specific investigation of critical short half-life infusion-interruption alarms, inclusion into the study required the presence of a clearly identifiable drug selection in one of the following groups: adrenaline/epinephrine, dobutamine, dopamine, or noradrenaline/norepinephrine.

### Exclusion Criteria

Pump profiles outside of critical care areas were excluded, as the absence of critical short half-life infusions in these areas risked distorting the volume of alarms against which clinicians must identify and prioritize critical short half-life infusion-interruption alarms. A simple *count* of whole hospital infusion alarm types was, however, included for comparative value.

### Data Manipulation and Interrogation

Data storage occurred via the CQI database—total data of approximately 60 GB—and the data were queried via SQL Server Management Studio v17.9.1 (Microsoft). Unique code was created for identification of specific pumps by count (see Multimedia Appendix 1, Table A1) and by significant alarm conditions for all pumps, as well as by profile (see Multimedia Appendix 1, Table A2). In order to review the total alarm count by profile across a large cross section of pumps from differing facilities, it may be required to carry out the interrogation given in Multimedia Appendix 1, Table A3, to identify the profile types.

The nomenclature used as a profile name or label may differ across organizations for the same *type* of critical care unit; this was the case in our sample group, as it was spread across the European region. For example, Table 3 shows those that were grouped under NICU.

**Table 3:** Profile names grouped under the neonatal intensive care unit (NICU).

Profile name	Profile ID
Neonatal	11
Neonates <2 kg	12
Neonates <2 kg	13
NEONATOLOGIE	3
NEONATOLOGIE	4
NICU	14
NICU 3	15
NICU rule of 6	16
SCBU^a^	79
SCN^b^	21

^a^SCBU: special care baby unit.

^b^SCN: special care nursery.

This grouping gave the profile ID numbers to be used for queries. The code used to interrogate the number of downstream occlusions in NICU is given in Multimedia Appendix 1, Table A4, as an example.

Where the nomenclature of the specific profiles used in the dataset and drug library are known, as would be the case in a single facility querying its data, the code given in Multimedia Appendix 1, Table A5, may be utilized. For pump count and pump type for specific profiles, the code given in Multimedia Appendix 1, Table A6, may be utilized.

The gross number of undifferentiated infusions per day was calculated from the CQI reporter software, which gives both total infusions started over the time period investigated and a breakdown of those started from within the drug library using the DERS. For the investigation of the number of alarms occurring specifically for critical short half-life infusion alarms, the various codes used are given in Multimedia Appendix 1, Table A7.

A manual review of the data obtained from the above code to exclude repeat alarms completed the analysis to allow for only the inclusion of *first-time* reaction time to infusion-interruption states and alarms to be recorded in the results of the study for the following:

Reaction time [in seconds] = (Time [hh:mm:ss] issue resolved and pump restarted) – (Time [hh:mm:ss] pump stopped and alarm triggered) (3)

## Results

Table 4 expresses the total and individual alarms and all infusion starts for each area of critical care and the whole hospital. From this, it can be identified that not only are the bulk of alarms seen in critical care, certainly due to the sheer number of infusions given in these areas, but there are also far more alarms per infusion in these areas too. Clearly the risk of alarm fatigue is greatest in these areas.

Infusion interruption of critical short half-life infusions was found to be a significant problem in all areas of the general critical care pump population with EOI and *end of syringe* events being noted, as well as a significant number of downstream (ie, vein and access) occlusion events. See Table 5 for a breakdown of critical short half-life infusion in the NICU by alarm type for each drug type. See Table 6 for a summary of restart times following alarms for the interruption of critical short half-life infusions.

**Table 4:** Infusion and alarm frequencies by care area and across the *whole hospital* between the study dates January 1, 2000, to September 29, 2017 (6482 days).

Parameter		Frequency in critical care areas, n					Frequency in *whole hospital*, n	Alarm frequency, %
		All	NICU^a^	GICU^b^	CCU^c^	PICU^d^		
Total infusion starts		187,441	30,527	45,756	110,254	904	1,600,832	N/A^e^
Alarms per infusion, mean		4.50	3.71	4.36	1.33	8.61	1.39	N/A
**Alarm types**								
	Total alarms	467,437	113,277	199,482	146,694	7784	2,211,457	100
	Flow error (ie, drip counter)	0	N/A	0	0	N/A	0	0
	Air-in-line accumulation exceeded	6975	N/A	6700	275	N/A	27,583	1.25
	Air-in-line single bubble exceeded	17,100	N/A	12,724	4376	N/A	108,701	4.92
	Callback	134,754	22,396	68,037	42,740	1581	802,691	36.30
	Door open while infusing	14	N/A	14	0	N/A	2710	0.12
	Drive engage failure	10,850	5629	175	4624	422	13,807	0.62
	End of infusion	20,762	4650	849	13,995	1268	23,903	1.08
	Near end of infusion	107,236	10,695	49,517	45,668	1356	278,969	12.61
	Occlusion (downstream)	136,148	62,944	49,444	21,314	2446	847,438	38.32
	Occlusion (upstream)	14,602	N/A	11,710	2892	N/A	86,592	3.92
	Syringe disengaged	15,400	6738	290	7915	457	15,288	0.69
	End of syringe	3396	225	22	2895	254	3775	0.17
Infusions started from within the drug library (ie, detectable as critical infusions), %		N/A	N/A	N/A	N/A	N/A	67.39	N/A

^a^NICU: neonatal intensive care unit.

^b^GICU: general intensive care unit.

^c^CCU: coronary care unit.

^d^PICU: pediatric intensive care unit.

^e^N/A: not applicable.

**Table 5:** Critical short half-life infusions and alarms in the neonatal intensive care unit (NICU).

Parameter		Frequency by drug type, n				Critical short half-life infusions and alarms for four drugs, n	Total infusion starts and alarms, n	Critical short half-life infusions and alarms for four drugs out of total infusion starts and alarms, %	Alarms for four drugs out of all critical short half-life infusion starts (N=3623), %
		DOB^a^	DA^b^	AD^c^	NAD^d^				
Total infusion starts		2509	96	58	960	3623	30,527	11.87	N/A^e^
**Alarm types**									
	Callback	2776	15	28	459	3278	22,396	14.64	90.48
	Drive engage failure	25	19	0	13	57	5629	1.01	1.57
	End of infusion	38	13	1	4	56	4650	1.20	1.55
	Near end of infusion	342	1	19	92	454	10,695	4.24	12.53
	Occlusion (downstream)	267	114	12	120	513	62,944	0.82	14.16
	Syringe disengaged	29	21	0	28	78	6738	1.16	2.15
	End of syringe	3	2	0	0	5	225	2.22	0.14

^a^DOB: dobutamine.

^b^DA: dopamine.

^c^AD: adrenaline.

^d^NAD: noradrenaline.

^e^N/A: not applicable.

**Table 6:** Time to restart following alarms for critical short half-life infusion interruptions.

Measure	Reaction times in seconds			
	Neonatal intensive care unit (NICU)	Pediatric intensive care unit (PICU)	General intensive care unit (GICU)	Coronary intensive care unit (CICU)
Mean (SD)	58.73 (43.63)	14.75 (7.25)	12.20 (22.58)	13.00 (17.24)
Maximum	444.25	24.75	29.50	63.00
Minimum	4.00	7.50	3.75	3.75
Median	17.50	12.75	7.50	8.38

## Discussion

Table 4 shows an initial analysis and a mean average of 4.50 alarms per infusion in the general critical care pump population. In the PICU, the alarms per infusion value was significantly above the mean average for all critical care areas, with 8.61 alarms per infusion. Both of these values are far above that seen in the general *whole hospital* pump population (ie, 1.39 alarms per infusion), which includes all pumps in critical care and all others in medical-surgical units, clinics, oncology centers, and all other care areas.

In terms of possible strategies for the mitigation of alarm fatigue, it is of value to review the two parameters NEOI and EOI. NEOI is an indicator to the clinician that an infusion is close to completion or close to empty in terms of the syringe or bag. NEOI was responsible for almost 13% of all infusion alarms (see Table 4) and occurred in 57% of all infusion starts. This is a large volume of alarms, which may or may not be significant; as discussed earlier, the alarm for NEOI, as for all alarms, is not differentiated between critical short half-life infusions and noncritical medications. Indeed, in the case of an intermittent drug such as an antibiotic, the clinician may in fact need to run the infusion until they empty the bag or syringe in order to give a full dose; in this case, the NEOI alarm is truly a nuisance alarm.

A centralized system for infusion monitoring can overcome much of this issue of alarm differentiation, provided that the medications are clearly identified as critical short half-life infusions or noncritical and are easily located or *mapped* from display to geographical location. While the alarm may continue, the clinician would be able to decide on the appropriate level of response.

For EOI and *end of syringe* alarms, the question of *rapid response* versus *standard response* very much depends on the question of whether the medication is a critical short half-life infusion or not. EOI and *end of syringe* alarms account for only 1% and 0.17% of all infusion alarms, respectively (see Table 4), and would therefore not contribute overly to alarm fatigue. However, these alarms are very clearly analogous to the problem of “crying wolf” identified by Waterson et al [12], in that they occur in a total of 1.7% of NICU critical short half-life infusions (see Table 5). For instance, they are rare events but potentially extremely hazardous and hard to detect and differentiate. This is the case, given the fact that EOI and *end of syringe* alarms are the same for all medications and are presented to the clinician among a large volume of alarms, even among the subgroup of critical short half-life infusions where 90.5% of these infusions had the noncritical alert Callback associated with them (see Table 5). Again, a centralized routed alarm management system with differentiated alarm tones and visual indicators for critical short half-life infusions would almost certainly be of value to reduce the “crying wolf” risk.

The ratio of *downstream occlusion* to *infusion starts* in the general pump population was 0.52; in the critical care areas it was 1.38, while in PICU and NICU it was 2.71 and 2.06, respectively (see Table 4). Given that extravasation injury remains the most common iatrogenic neonatal injury [13], it is clear that these occlusion alarms are not merely nuisance alarms and that prompt attendance to them, followed by inspection of the intravenous cannula site and appropriate action, may prevent worsening of any infiltration or extravasation injury [14]. They are *urgent alarms* and they are also as likely as critical short half-life interruption alarms to be missed among the white noise of the mass of alarms to which clinicians are exposed. Any centralized or distributed alarm system employed to ensure rapid and appropriate response must be capable of indicating clearly the particular problem to be addressed by the clinician. Such a system should also be capable of indicating parameters such as vein pressure to allow clinicians to become proactive in infusion management. This study indicates how capturing infusion data from right across the health care facility makes it possible to identify problematic areas of the hospital for potential alarm fatigue as well as particular issues of infusion and infusion line management in specific areas.

**Figure 2:**
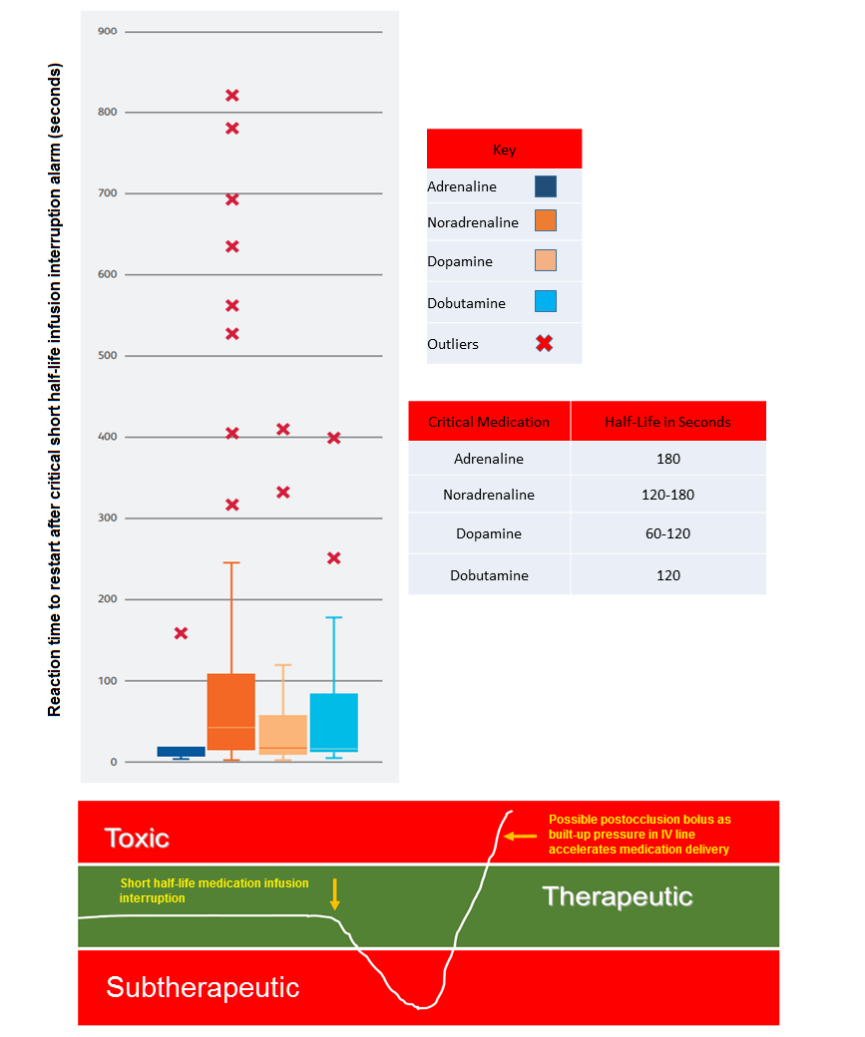
Restart reaction times after critical short half-life infusion interruption alarms in the neonatal intensive care unit (NICU): means (horizontal lines within the boxes), quartiles (upper and lower edges of the boxes), and outliers (red Xs). Also shown are indicative figures for consequences of prolonged interruption of critical short half-life infusions and the half-lives of critical medications in seconds. IV: intravenous.

The mean and median response times to critical short half-life infusion interruption were generally within the half-lives of the selected medications (see Table 6). However, there was a high prevalence of outliers in terms of reaction times for all the critical short half-life infusions studied, with some response times being longer than 13 minutes or 6 times greater than the accepted plasma half-life of the concerned medication (see Figure 2).

Escalation of alarms from pumps that may be in isolation rooms or simply in geographically dispersed locations has been shown to be of value in one study where central infusion monitoring was introduced to an NICU along with environmental changes. A 56.25% reduction in measured infusion alarms was recorded in the study as well as a 31% reduction in reaction time to infusion alarms for critical short half-life infusions [15].

With the trend toward increasing numbers of satellite critical care and high-dependency beds, more isolation rooms, and increased numbers of *chronic critical care* patients, the number of complex intravenous infusion therapies given in dispersed locations is likely to increase. The need for strategies and technologies to manage the risk of alarm fatigue and prolonged interruption of critical short half-life infusions has been identified in this study, along with particular issues of infusion management that warrant further attention and appropriate interventions. Indeed, pump data have been previously used to help inform and support intervention planning, such as added training and resetting default pump alarm settings, by targeting specific problems such as downstream occlusions and specific care areas based on the data pulled from pumps [2]. These interventions only produced a 2% reduction in alarms per patient per day. It was felt that this limited impact was due to the fact that the project was limited in its flexibility, as “weekly adjustments to the process” were not realistic given the time required for data extraction and analysis; as well, the project was limited geographically, as the project could only be completed in one hospital unit [2].

This study lays out baseline data for possible benchmarking from units right across the hospital. It also has the flexibility and speed of application through established SQL (structured query language) query codes to allow for rapid and comprehensive data processing, allowing for rapid analysis of change pre- and post- any intervention. The accumulation of data can, of course, also be expected to increase with networked pumps, particularly if wireless pumps are deployed to dispersed locations within the hospital. Such an accumulation of data would allow for a deeper look into noncritical care areas where alarm management might benefit from an extension of alarm notification beyond central monitoring to *clients* in the form of tablet computers or other handheld devices.

One clear conclusion from this study is that the medication administration *rights* [16] should be extended to include *right maintenance* and ensured delivery continuity of critical short half-life infusions. There has been much useful focus in medication safety on ensuring correct and safe initial setup of intravenous medication infusions. However, without a focus on ensuring continuous and uninterrupted delivery of infusions, particularly in the case of short half-life medications, much of the effectiveness of these setup strategies is essentially redundant.

## Appendix

Multimedia Appendix 1 Microsoft SQL Server Management Studio v17.9.1 query codes. SQL: structured query language.

## Abbreviations

AD: adrenaline
BD: Becton, Dickinson and Company
CCU: coronary care unit
CICU: coronary (adult) intensive care unit
CQI: continuous quality improvement
DA: dopamine
DERS: dose error reduction software
DOB: dobutamine
EOI: end of infusion
GICU: general (adult) intensive care unit
GTN/NTG: glycerol-trinitrate/nitroglycerine
ICN: intensive care nursery
ICU: intensive care unit
LVP: large volume pump
NAD: noradrenaline
NEOI: near end of infusion
NICU: neonatal intensive care unit
PICU: pediatric intensive care unit
SCBU: special care baby unit
SCN: special care nursery
SP: syringe pump

## References

[1] Cho OM, Kim H, Lee YW, Cho I (2016). Clinical alarms in intensive care units: Perceived obstacles of alarm management and alarm fatigue in nurses. Healthc Inform Res.

[2] Matocha D (2018). Reducing infusion pump alarms through structured interventions. J Assoc Vasc Access.

[3] Rothschild J, Landrigan C, Cronin J, Kaushal R, Lockley S, Burdick E, Stone P, Lilly C, Katz J, Czeisler C, Bates D (2005). The Critical Care Safety Study: The incidence and nature of adverse events and serious medical errors in intensive care. Crit Care Med.

[4] Sowan AK, Gomez TM, Tarriela AF, Reed CC, Paper BM (2016). Changes in default alarm settings and standard in-service are insufficient to improve alarm fatigue in an intensive care unit: A pilot project. JMIR Hum Factors.

[5] Lee P, Thompson F, Thimbleby H (2012). Analysis of infusion pump error logs and their significance for health care. Br J Nurs.

[6] Yu D, Hsu K, Kim JH, DeLaurentis P (2017). Infusion pump informatics approach to quantify impact of alerts and alarms on healthcare delivery. Proceedings of the Human Factors and Ergonomics Society Annual Meeting.

[7] Waterson J (2013). Making smart pumps smarter, making IV therapy safer. Br J Nurs.

[8] Neff T, Fischer J, Fehr S, Baenziger O, Weiss M (2001). Evaluation of the FASTSTART mode for reducing start-up delay in syringe pump infusion systems. Swiss Med Wkly.

[9] Quinn C (2000). Infusion devices: Risks, functions and management. Nurs Stand.

[10] KLAS Research.

[11] Bergon-Sendin E, Perez-Grande C, Lora-Pablos D, Moral-Pumarega MT, Melgar-Bonis A, Peña-Peloche C, Diezma-Rodino M, García-San Jose L, Cabañes-Alonso E, Pallas-Alonso CR (2015). Smart pumps and random safety audits in a neonatal intensive care unit: A new challenge for patient safety. BMC Pediatr.

[12] Waterson J, Garcia S, Vollmer C, Rolt S (2018). Crying wolf: Alarm fatigue and response times for vasoactive infusions. Proceedings of the 8th International Congress of the Union of European Neonatal and Perinatal Societies (UENPS).

[13] Reynolds BC (2007). Neonatal extravasation injury: Case report. Infant.

[14] Hadaway LC (2004). Preventing and managing peripheral extravasation. Nursing.

[15] Bastanie M, Blom H, Mahieu L, Meeus M, Laroche S, VanLaere D, Voeten M, Demeulemeester V, Vermeulen R, Mulder A (2017). Alarm visibility and infusion continuity: Environmental change and technology assistance. Proceedings of the 2nd Congress of Joint European Neonatal Societies (jENS 2017).

[16] (2007). Institute for Safe Medication Practices (ISMP).

