# Supplementary material for: Types and Frequency of Infusion Pump Alarms and Infusion-Interruption to Infusion-Recovery Times for Critical Short Half-Life Infusions: Retrospective Data Analysis
Source: JMIR Hum Factors. 2019 Aug 12;6(3):e14123. doi: 10.2196/14123 (PMC6709565; doi:10.2196/14123)
Supplement: Multimedia Appendix 1 [file humanfactors_v6i3e14123_app1.pdf]

Appendix: Microsoft SQL Server Management Studio v17.9.1 Query Codes.

Table A1: Pump Type Count via SQL Query.

Pump Type	Code SQL Query
Volume Pump Count:	SELECT COUNT ('pump_type_id') FROM CQI.PumpInfo WHERE pump_type_id IN (2,3)
Syringe Pump Count:	SELECT COUNT ('pump_type_id') FROM CQI.PumpInfo WHERE pump_type_id IN (0,1)

Table A2: SQL Queries for all Alarm Data.

Alarm Type Across All Pumps	Code SQL Query
Callback	SELECT COUNT ('event_code') FROM CQI.EventInfo WHERE event_code IN ('CALLBACK')
Drive Engage Failure	SELECT COUNT ('event_code') FROM CQI.EventInfo WHERE event_code IN ('DRVDISNG')
End of Infusion	SELECT COUNT ('event_code') FROM CQI.EventInfo WHERE event_code IN ('EOI')
Near End of Infusion	SELECT COUNT ('event_code') FROM CQI.EventInfo WHERE event_code IN ('NEOI')
Occlusion (Down Stream)	SELECT COUNT ('event_code') FROM CQI.EventInfo WHERE event_code IN ('OCCL')
Syringe Disengaged	SELECT COUNT ('event_code') FROM CQI.EventInfo WHERE event_code IN ('SYRDISNG')
End of Syringe	SELECT COUNT ('event_code') FROM CQI.EventInfo WHERE event_code IN ('ENDOFSYR')
Air In Line Accumulation Exceeded [Volume Pump Only]	SELECT COUNT ('event_code') FROM CQI.EventInfo WHERE event_code IN ('AILACCUM')
Air In Line Single Bubble Exceeded [Volume Pump Only]	SELECT COUNT ('event_code') FROM CQI.EventInfo WHERE event_code IN ('AILSINGLE')
Door Open While Infusing [Volume Pump Only]	SELECT COUNT ('event_code') FROM CQI.EventInfo WHERE event_code IN ('DOOROPEN')
Occlusion (Up Stream) [Volume Pump Only]	SELECT COUNT ('event_code') FROM CQI.EventInfo WHERE event_code IN ('UPOCCL')

Table A3: Identification of Profile Types:

Identification of Profile/ Care Area type	Code SQL Query
Profile Name with Profile ID Number	SELECT profile_name,profile_id FROM CQI.profile

Table A4: Example of Profile ID Numbers being utilized for Queries. Interrogating the Number of Downstream Occlusions in NICU.

```
SELECT COUNT ('event_code') FROM CQI.EventInfo
WHERE event_code IN ('OCCL') AND profile_id IN (11,12,13,3,4,14,15,16,79,21)
```

Table A5: Code for Dataset Interrogation where the Nomenclature of Specific Profiles used in the Dataset and Drug Library are Known.

```
SELECT COUNT ('event_code') FROM CQI.EventInfo
WHERE event_code IN ('EVENT') AND profile_name IN ('NOMENCLATURE')
```

Table A6: Code for Pump Count and Pump Type for Specific Profiles.

```
SELECT COUNT (Distinct t2.pump_id) FROM CQI.EventInfo as t1, CQI.PumpInfo as t2
WHERE t2.pump_type_id IN(-,-)
AND t1.profile_id in (-,-,-,-,-)
AND t1.pump_id = t2.pump_id
AND t1.event_code IN ('PWRUP')
```

Table A7: SQL Codes giving the number of alarms occurring specifically for critical short half-life infusion alarms.

```
-- database to be used name:

USE CQI

SELECT COUNT ('event_id')
FROM CQI.EventInfo s0

JOIN CQI.Drug ON s0.drug_id = Drug.drug_id

-- wildcard for drug name:
WHERE drug_name LIKE '%DOPA%'

-- list of alarms to be used:
AND event_code IN ('START','OCCL','SYRDISNG','DRVDISNG','ENDOFSYR','EOI','NEOI')
-- AND event_code IN
('OCCL','SYRDISNG','DRVDISNG','ENDOFSYR','EOI','NEOI','AILACCUM','AILSINGLE','CALLBA
CK','DOOROPEN','FLOWERR','UPOCCL')
-- AND event_code IN ('OCCL','SYRDISNG','DRVDISNG','ENDOFSYR','EOI','NEOI')
-- AND event_code IN
('AILACCUM','AILSINGLE','CALLBACK','DOOROPEN','FLOWERR','UPOCCL')
```

With replacement of '%DOPA%' with '%NOR%', '%DOBU%', '%EPI%', '%ADRE%' For each critical short half-life infusion to be assayed.

In order to exclude the possibility of anomalies, with the introduction of spurious drugs the following code can be run with the same substitutions as above:

```
-- database to be used name:

USE CQI

SELECT event_id, timestamp, event_code, data_set_id, profile_id, s0.drug_id,
drug_name, pump_id, pump_info_id
FROM CQI.EventInfo s0

JOIN CQI.Drug ON s0.drug_id = Drug.drug_id

-- wildcard for drug name:
WHERE drug_name LIKE '%DOPA%'

-- list of alarms to be used:
AND event_code IN ('START','OCCL','SYRDISNG','DRVDISNG','ENDOFSYR','EOI','NEOI')
-- AND event_code IN
('OCCL','SYRDISNG','DRVDISNG','ENDOFSYR','EOI','NEOI','AILACCUM','AILSINGLE','CALLBA
CK','DOOROPEN','FLOWERR','UPOCCL')
-- AND event_code IN ('OCCL','SYRDISNG','DRVDISNG','ENDOFSYR','EOI','NEOI')
-- AND event_code IN
('AILACCUM','AILSINGLE','CALLBACK','DOOROPEN','FLOWERR','UPOCCL')
```

Replacement of the code section:

```
-- wildcard for drug name:
WHERE drug_name LIKE '%DOPA%'
```

With:

```
-- wildcard for drug name:
WHERE drug_name LIKE '%DOPA%' AND profile_id IN (KNOWN PROFILE IDS)
```

e.g.

```
-- wildcard for drug name:
WHERE drug_name LIKE '%DOPA%' AND profile_id IN (11,12,13,3,4,14,15,16,79,21)
```

Or use

```
AND profile_id IN (‘KNOWN NOMENCLATURE)
```

In the above the codes allow interrogation of specific profiles for the critical short half-life infusion alarm count and types of alarm by profile.

For the investigation of infusion interruption alarms occurring specifically for critical short half-life infusion and reaction times of clinicians to these alarms the following code can be applied:

```
-- database to be used name:
USE CQI
```

```sql
GO

-- define maximum time difference in seconds:
DECLARE @timeDiff INT = 3600;

SELECT s0.event_id,s0.timestamp,s0.event_code,
              s1.event_id,s1.timestamp,s1.event_code,
              -- s1.data_set_id, s1.profile_id, s1.drug_id, s1.pump_id,
              drug_name,
              DATEDIFF(SECOND,s0.timestamp,s1.timestamp) as 'Time difference (s)'
              ,s0.profile_id, Profile.profile_name
FROM cqi.EventInfo s0, cqi.EventInfo s1

JOIN CQI.Drug ON s1.drug_id = Drug.drug_id
JOIN CQI.Profile ON s1.profile_id = Profile.profile_id

-- syringe alarms:
-- WHERE s0.event_code IN ('OCCL','SYRDISNG','DRVDISNG')
-- WHERE s0.event_code IN ('OCCL','SYRDISNG','DRVDISNG','ENDOFSYR','EOI')
-- volumetric alarms:
-- WHERE s0.event_code IN ('AILACCUM','AILSINGLE','CALLBACK','DOOROPEN','FLOWERR','UPOCCL')
-- syringe and volumetric alarms:
WHERE s0.event_code IN
('OCCL','SYRDISNG','DRVDISNG','ENDOFSYR','EOI','NEOI','AILACCUM','AILSINGLE','DOOROPEN','FL
OWERR','UPOCCL')

AND s1.event_code ='START'
AND s1.event_id = s0.event_id+1
AND DATEDIFF(minute,s0.timestamp,s1.timestamp) >= 0
-- if maximum time difference between the events is required:
-- AND DATEDIFF(minute,s0.timestamp,s1.timestamp) <= @timeDiff
AND s0.pump_id = s1.pump_id
AND s0.data_set_id = s1.data_set_id
AND s0.profile_id = s1.profile_id

-- wildcard for drug name:
AND drug_name LIKE '%nor%'
-- AND drug_name LIKE '%dop&'
-- AND drug_name LIKE '%dob&'
-- AND drug_name LIKE '%adre&'

-- results sorted by time difference:
-- ORDER BY 'Time difference (s)'

-- results sorted by profile_id
ORDER BY profile_id
```
